# A Bayesian Dynamical Approach for Human Action Recognition

**DOI:** 10.3390/s21165613

**Published:** 2021-08-20

**Authors:** Amirreza Farnoosh, Zhouping Wang, Shaotong Zhu, Sarah Ostadabbas

**Affiliations:** Augmented Cognition Lab (ACLab), Department of Electrical and Computer Engineering, Northeastern University, Boston, MA 02115, USA; afarnoosh@ece.neu.edu (A.F.); zpwang@ece.neu.edu (Z.W.); shawnzhu@ece.neu.edu (S.Z.)

**Keywords:** 3D skeletal motion, bayesian inference, biologically valid interpretation, deep generative models, human action recognition, latent state modeling, motion capture, switching dynamical modeling, variational inference

## Abstract

We introduce a generative Bayesian switching dynamical model for action recognition in 3D skeletal data. Our model encodes highly correlated skeletal data into a few sets of low-dimensional switching temporal processes and from there decodes to the motion data and their associated action labels. We parameterize these temporal processes with regard to a switching deep autoregressive prior to accommodate both multimodal and higher-order nonlinear inter-dependencies. This results in a dynamical deep generative latent model that parses meaningful intrinsic states in skeletal dynamics and enables action recognition. These sequences of states provide visual and quantitative interpretations about motion primitives that gave rise to each action class, which have not been explored previously. In contrast to previous works, which often overlook temporal dynamics, our method explicitly model temporal transitions and is generative. Our experiments on two large-scale 3D skeletal datasets substantiate the superior performance of our model in comparison with the state-of-the-art methods. Specifically, our method achieved 6.3% higher action classification accuracy (by incorporating a dynamical generative framework), and 3.5% better predictive error (by employing a nonlinear second-order dynamical transition model) when compared with the best-performing competitors.

## 1. Introduction

Analyzing 3D motion capture datasets, illustrating dynamical motions of a subject and inferring their actions is the key processing step in many applications, including highlighting movement patterns of an athlete to optimize their performance, probing behavior of an endangered animal, and monitoring mobility of a patient in a rehabilitation study, to name a few [1]. In all these applications, body pose data contained in the motion capture sequence describes temporal evolution of specific phenomena or action and is switching between potentially limited number of states each representing a specific regime.

The efforts in quantifying complex kinematics of biological mechanisms in a lower dimensional subspace have led to the successful design of bio-inspired robots that can mimic their biological counterparts to a great extent [2,3,4]. For instance, it is shown that more than 80% of the variance of static grasping data in humans could be described by the first two postural synergies [5]. Skeleton-based human action recognition, on the other hand, has been extensively studied in the context of deep learning using convolutional neural networks [6,7], recurrent neural networks [8,9], and graph convolutional networks [10,11,12].

However, there are still some limitations with the existing body of works. (I) A vast majority of previous state-of-the art models perform action classification on motion segments that only include a single action. In other words, they predict a single action label for the entire motion segment. This substantially decreases their applicability in practice for real-world applications because in most cases (if not all) we are not given single-action segments and the action recognition model has to determine action segments itself. A practical algorithm should be able to provide per-time point action labels and update these labels accordingly in real-time. (II) Although some previous works capture temporal correlations in motion data (e.g., through temporal smoothness regularization), there are very few methods that explicitly model temporal dynamics/transitions. For the task of human action recognition, most of the existing models are deterministic, specifically designed for action recognition, and inherently do not model temporal dynamics or learn data distribution, and therefore are not generative. (III) Most of the previous methods cannot handle missing entries in motion data (which may happen frequently) because they are not predictive. Their common practice is to simply set missing entries to zero or remove their corresponding time points entirely. (IV) Most of the previous works do not provide any interpretations or intuitions for their predictions of action labels in terms of intrinsic dynamical processes in motion data or motion primitives.

**Figure 1:**
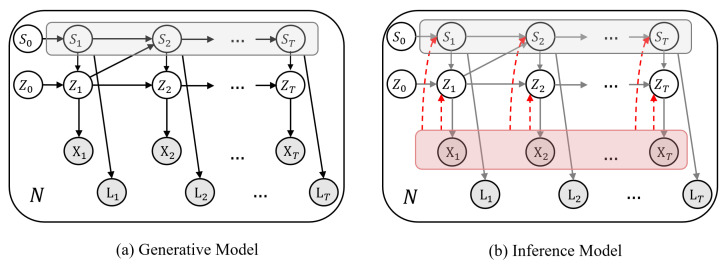
(**a**) Graphical representation of our generative model given *N* motion datasets $X_{n}$ and their action labels $L_{n}$. The low-dimensional continuous latents $Z_{n}={\left\{Z_{n,t}\right\}}_{t=1}^{T}$ are generated with regards to a nonlinear autoregressive prior switched by their associated discrete states $S_{n}={\left\{S_{n,t}\right\}}_{t=1}^{T}$. The discrete states themselves are determined according to a Markovian prior conditioned on their preceding continuous latents, i.e., $Z_{t-1}$. (**b**) In the inference model, discrete and continuous latents are estimated from motion sequences $X_{n}$ in an amortized fashion (denoted by dashed red arrows).

**Figure 2:**
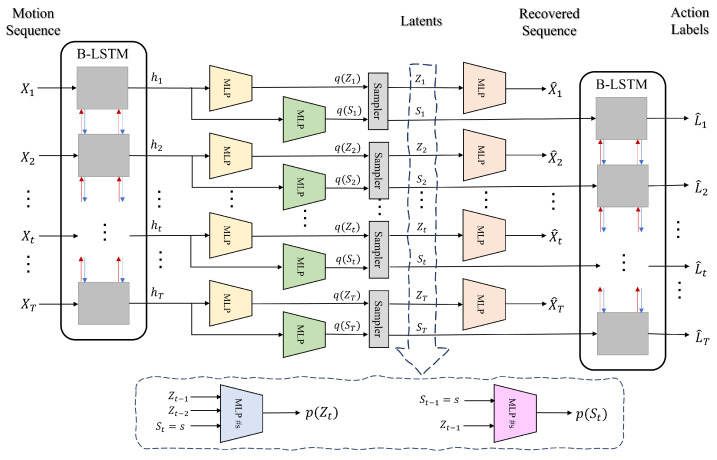
Visual framework of our proposed method. Our model encodes an input motion sequence $\left\{X_{1},\cdots ,X_{T}\right\}$ into a sequence of hidden features $\left\{h_{1},\cdots ,h_{T}\right\}$ using a bidirectional LSTM. The resulting hidden features are fed to two separate MLPs for estimating variational distribution parameters of discrete latents $\left\{S_{1},\cdots ,S_{T}\right\}$ and continuous latents, $\left\{Z_{1},\cdots ,Z_{T}\right\}$. These posterior distributions are then sampled to obtain their latent values. We decode to the input motion sequence $\left\{{\hat{X}}_{1},\cdots ,{\hat{X}}_{t}\right\}$ by feeding continuous latents *Z* to an MLP. We also decode to the associated action labels $\left\{{\hat{L}}_{1},\cdots ,{\hat{L}}_{T}\right\}$ by feeding probability vectors of the discrete latents S to a bidirectional LSTM. We estimate the priors for the discrete latents $p(S_{t})$ and continuous latents $p(Z_{t})$ from the values of sampled latents using two separate MLPs.

The strong spatio-temporal correlation among joints of a human’s skeleton captured by 3D motion capture data as well as its clear sparseness motivate the utilization of dynamical probabilistic models that can learn underlying interpretable states from data and extract their low-dimensional motion patterns. To this end, we propose a Bayesian state switching model for dynamical action recognition that is both generative and predictive. Specifically, we employ a low-dimensional deep generative latent model to encode highly correlated skeleton data into a few set of switching autoregressive temporal processes. This model then decodes from these low-dimensional representations to the skeletal data and their associated action labels. This results in a flexible model that accommodates multimodal and higher-order nonlinear inter-dependencies, parses meaningful temporal modes in 3D pose data, and enables action recognition. Specifically, we make the following contributions:

- In contrast to previous works which merely classify motion sequences into action labels and are not generative, our method, sketched in Figure 1 and Figure 2, in addition to action recognition, (I) segments motion data from a dynamical perspective by explicitly modeling temporal dynamics in a Bayesian approach, hence, (II) it allows dynamical prediction of skeletal motion from low-level representations. Specifically, (III) it welcomes multimodal, higher-order, and nonlinear temporal relations in motion data by employing a deep switching auto-regressive latent model.
- Our method can easily fill in missing entries in motion data due to its predictive nature which models transitions between time points and between action labels.
- Our method provides action labels per time point and can handle varying-length sequences.
- Our method uses a nonlinear second-order dynamical model to better capture skeletal dynamics, because first-order models are less effective in modeling acceleration (i.e., second-derivative of location) in motion data.
- The sequence of discrete latents in our method enables multi-modal dynamical modeling and at the same time provides visual/qualitative and quantitative interpretations about motion primitives that gave rise to each action class, which were not possible with previous methods.
- Our method is probabilistic and provides confidence intervals for its estimations and predictions.

We demonstrate superior performance of our model on two large-scale 3D skeletal motion datasets in terms of action classification and dynamical prediction accuracy. The source code is available at github.com/ostadabbas/ActionDSARF (accessed on 15 August 2021).

## 2. Related Works

Here, we give a short overview of the recent advancements in dynamical system modeling as well as human action recognition using skeletal data, two fundamental aspects of our proposed approach.

### 2.1. Dynamical Systems Modeling

Switching linear dynamical systems (SLDS) have long been investigated in the literature [13,14,15,16,17,18]. These models decompose time series data into series of simpler, repeated dynamical modes represented by discrete and continuous latent states. In SLDS framework, the transitions between discrete states are independent of their associated continuous values. This problem is addressed in recurrent switching linear dynamical system (rSLDS) [19,20,21], by allowing the discrete state transition probabilities to depend on their preceding continuous states. The rSLDS dynamical capacity is however limited as it assumes first-order linear dynamics. A recent work [22], extends these models by adopting nonlinear and higher-order multimodal dependencies through a deep switching autoregressive framework. We build our model on top of this framework and customize that for dynamical action recognition. This makes our model flexible for complex auto-regressive relations in motion sequences.

From another perspective, dynamical matrix factorization is used in [23,24,25,26,27] for modeling linear dynamics in their low-dimensional temporal factors. Several studies have also employed neural networks for non-linear state-space modeling [28,29,30,31,32,33], which are restricted to first-order Markovian dependencies, and for time series prediction [34,35,36,37,38,39], most of which are non-probabilistic.

### 2.2. Action Recognition

Skeleton-based human action recognition has drawn much attention for its efficiency and robustness compared to the image-based action recognition methods. Earlier works [40,41] treat joint coordinates as independent features and model the relationship between them through hand-crafted feature vectors. After the introduction of deep learning, both convolutional and recurrent networks are used to extract information from skeleton data after transforming them into pseudo images or a sequence of vectors. In order to accomplish this transformation both spatially and temporally, Ref. [6] proposed their joint trajectory maps to represent spatial configuration and joint dynamics using color-coded texture images. Authors in [7] proposed the shape-motion representation from geometric algebra, which addressed the importance of both joints and bones and fully utilized the information provided by skeleton sequence. Recurrent neural network (RNN) structure can effectively process sequential data, while it has more difficulties for spatial information modeling. A novel two-stream RNN architecture to model both temporal dynamics and spatial configurations for skeleton data is proposed in [8]. Human actions can also be interpreted based on the interactions between body parts. In [9], authors proposed part-aware long short-term memory (P-LSTM), which divides the entire body dynamics based on different body parts and learn the final classifier over their concatenation. Common temporal patterns of the parts are learnt independently and then combined in the global level representation for action recognition. Recent approaches model the joint movements of an action with skeleton spatial-temporal graphs. Authors in [10] proposed to model the skeleton data with the graph convolutional networks (GCNs), in which the spatial graph convolution and the temporal graph convolution are both utilized. Spatial edges correspond to the connection of joints at each frame and the temporal edges connect the same joints across frames. In this configuration, the spatio-temporal information can be extracted based on the multi-layer graph convolution. To extract discriminative spatio-temporal features more effectively, [11] proposed a novel graph-based LSTM network to capture discriminative features by exploring the co-occurrence relationship between spatial and temporal spaces. Hierarchical architectures are also used to increase the temporal receptive fields. In [12], the skeleton data are represented as a directed acyclic graph (DAG) based on the kinematic dependency between the joints and bones in the natural human body to combine these two types of data for a better usage. In order to unbiasedly model long-range joint relationship under multi-scale operators and get unobstructed cross space-time information flow for capturing complex spatial-temporal dependencies, Ref. [42] proposed multi-scale aggregation scheme disentangling the importance of nodes in different neighborhoods for effective long-range modeling.

More recently, Ref. [43] proposed a bi-directional long short-term memory (BiLSTM) based attention mechanism with a dilated convolutional neural network (DCNN) for human action recognition in videos. They used DCNN layers equipped with residual blocks to extract discriminative features from video frames. These features are then fed into a BiLSTM network to learn temporal dependencies, which is followed by an attention mechanism to perform action classification. Also, for the task of speech emotion recognition, Ref. [44] proposed a two-stream deep convolutional neural network with an iterative neighborhood component analysis (INCA) to learn refined spatiotemporal features. They used two distinct convolutional neural networks, denoted as two streams, to extract spatial and spectral features separately. These features are then combined and fed to an INCA for further refinement. Finally, the jointly refined features are passed from a fully connected network with a softmax classifier to predict emotion categories.

## 3. Problem Formulation

We consider a set of *N* motion datasets $\left\{X_{1},\cdots ,X_{N}\right\}$ and their associated action labels $\left\{L_{1},\cdots ,L_{N}\right\}$, where each $X_{n}\in R^{T\times (D\times 3)}$ contains 3D coordinates of *D* skeleton joints over *T* time points and $L_{n}\in {\left\{1,\cdots ,A\right\}}^{T}$ denotes its per-time point action labels from a set of *A* action classes. We propose a low-dimensional deep latent model that learns the generative distribution of these data and infers their latent representations as well as actions labels for an unseen sequence, which we will explain below as “Generative” and “Inference” models, respectively.

### 3.1. Generative Model

We assume that each data pair $\left\{X_{n},L_{n}\right\}$ is generated according to a set of *discrete* latent states $S_{n}={\left\{S_{n,t}\right\}}_{t=1}^{T}$ representing motion primitives and their corresponding low-dimensional *continuous* temporal latent variables $Z_{n}={\left\{Z_{n,t}\in R^{K}\right\}}_{t=1}^{T}$:

(1) $$\begin{array}{cc} X_{n} & ∼p_{\theta }\left(X_{n}\;|\;Z_{n}\right),\\ L_{n} & ∼p_{\theta }\left(L_{n}\;|\;S_{n}\right),\\ Z_{n} & ∼p_{\theta }\left(Z_{n}\;|\;S_{n}\right),\\ S_{n} & ∼p_{\theta }\left(S\right), \end{array}$$

where $p_{\theta }\left(X_{n}|Z_{n}\right)$ and $p_{\theta }\left(L_{n}|S_{n}\right)$ are emission distributions that define conditional probabilities of observation space for motion data $X_{n}$ and their action labels $L_{n}$ with respect to local continuous latents $Z_{n}$ and discrete latents $S_{n}$, respectively. $p_{\theta }\left(Z_{n}|S_{n}\right)$ is a switching dynamical autoregressive prior over $Z_{n}$ and $p_{\theta }\left(S\right)$ is a generative Markovian prior over $S_{n}$. We parameterize all distributions with neural networks and collectively denote their generative model parameters by θ. The graphical representation for our proposed generative model is depicted in Figure 1a.

#### 3.1.1. Discrete Markovian Prior $p_{\theta }\left(S\right)$

We construct a predictive dynamical framework to capture temporal coherence in our sequential data. We assume that this dynamical generative process resides at a specific state at each time point (out of *S* possible states) which is determined according to a Markovian prior conditioned on its preceding discrete latent $S_{t-1}$ and continuous latent $Z_{t-1}$. As such, the discrete latent states $S_{n}={\left\{S_{n,t}\right\}}_{t=1}^{T}$ are structured in a Markov chain as follows:

(2) $$p_{\theta }\left(S_{t}|S_{t-1}=s,Z_{t-1}\right)=\mathrm{Cat}\left(\sigma \left(\Phi_{\theta }^{s}\left(Z_{t-1}\right)\right)\right),$$

where $\Phi_{\theta }^{s}\left(\cdot \right)$ is a state-specific mapping parameterized by neural networks and σ(·) is a softmax activation function that ensures a valid *S*-dimensional probability vector. As noted in [19], conditioning the discrete states on their preceding continuous latents (in addition to their preceding discrete states) is desirable as it allows informed transitions.

#### 3.1.2. Switching Autoregressive Prior $p_{\theta }\left(Z_{n}|S_{n}\right)$

We assume that the low-dimensional continuous latents $Z_{n}$ follow a nonlinear autoregressive Gaussian prior switched by their associated discrete states $S_{n}$. This implies a Gaussian mixture distribution for the dynamical latent space:

(3) $$\begin{array}{c} p_{\theta }\left(Z_{t}|Z_{t-\ell },S_{t}=s\right)=\mathrm{Norm}\left(\mu_{\theta }^{Z,s}\left(Z_{t-\ell }\right),\sigma_{\theta }^{Z,s}\left(Z_{t-\ell }\right)\right), \end{array}$$

where s∈{1,⋯,S} and *ℓ* denotes a lag set (e.g., ℓ={1,2} for a second-order Markov model), and state-specific $\mu_{\theta }^{Z,s}\left(\cdot \right)$ and $\sigma_{\theta }^{Z,s}\left(\cdot \right)$ are parameterized by multilayer perceptrons (MLPs) (see Table 1). In other words, we feed $Z_{t-\ell }$ to a multi-head MLP for estimation of the Gaussian parameters, e.g., $\mu_{\theta }^{Z,s}\left(Z_{t-\ell }\right)=\sum_{l\in \ell }{\mathrm{MLP}}_{\theta }^{s,l}\left(Z_{t-l}\right)$.

#### 3.1.3. Emission Model for Action Labels $p_{\theta }\left(L_{n}|S_{n}\right)$

We specify a categorical emission model to determine action labels at each time point from the sequence of discrete state latents $S_{1:T}$. To this end, we summarize state latents at each time point into a hidden vector $h_{t}^{S}$ using a bidirectional LSTM recurrent network:

$$\begin{array}{cc} p_{\theta }\left(L_{t}|S_{1:T}\right) & =\mathrm{Cat}\left(\sigma \left(\Phi_{\theta }^{L}\left(h_{t}^{S}\right)\right)\right),\\ h_{1:T}^{S} & =B-\mathrm{LSTM}(S_{1:T}), \end{array}$$

where $\Phi_{\theta }^{L}\left(\cdot \right)$ is a neural network mapping. This modeling framework implies that an action label is decided only after observing its preceding and succeeding state latents in time.

#### 3.1.4. Emission Model for Motion Sequence $p_{\theta }\left(X_{n}|Z_{n}\right)$

We consider a Gaussian emission distribution for the observed motion instance $X_{t}$ conditioned on its continuous latent $Z_{t}$. Namely, the mean of this Gaussian is a function of the continuous latent value:

(4) $$\begin{array}{c} p_{\theta }\left(X_{t}|Z_{t}\right)=\mathrm{Norm}\left(\mu_{\theta }^{X}\left(Z_{t}\right),\sigma^{X}I\right), \end{array}$$

where $\mu_{\theta }^{X}\left(\cdot \right)$ is a nonlinear mapping parameterized by a neural network and $\sigma^{X}$ denotes observation noise.

### 3.2. Inference Model

As the posterior probability for this model is intractable, we use approximate variational methods in the form of amortized inference to learn the model parameters [45,46]. These methods approximate the posterior of latents $p_{\theta }\left(S,Z\;|\;X,L\right)$ with a variational distribution $q_{\phi }\left(S,Z\;|\;X\right)$ by maximizing the evidence lower bound (ELBO):

(5) $$\begin{array}{cc} L(\theta ,\phi )= & \;E_{q_{\phi }\left(S,Z|X\right)}\left[\log\frac{p_{\theta }\left(X,L,S,Z\right)}{q_{\phi }\left(S,Z|X\right)}\right]\\ = & \log p_{\theta }\left(X,L\right)-\mathrm{KL}\left(q_{\phi }\left(S,Z|X\right)\;∥\;p_{\theta }\left(S,Z|X,L\right)\right). \end{array}$$

By maximizing ELBO with respect to the parameters θ, we learn a generative model that defines a distribution over datasets pairs $p_{\theta }\left(X,L\right)$. By maximizing ELBO over the variational parameters ϕ, we perform Bayesian inference. Here, we assume a factorized variational distribution for the latents {S,Z}:

(6) $$q_{\phi }\left(S,Z|X\right)=\prod_{n=1}^{N}\prod_{t=1}^{T}q_{\phi }\left(S_{n,t}|X\right)q_{\phi }\left(Z_{n,t}|X\right),$$

where its distribution parameters are estimated from input motion sequences. The graphical representation for the proposed inference model is depicted in Figure 1.

#### 3.2.1. Variational Distributions for Discrete and Continuous Latents $q_{\phi }\left(S_{n,t}|X\right)$, $q_{\phi }\left(Z_{n,t}|X\right)$

For discrete state latents $S_{1:T}$, we specify a categorical variational distribution at each time point, whose parameter vector is a function of observed motion sequence. For continuous latents $Z_{1:T}$, we assume a Gaussian variational distribution at each time point, whose mean and covariance are functions of observed motion sequence. To this end, we encode each motion sequence into a hidden vector $h_{t}^{X}$ at each time point using a bidirectional LSTM recurrent network:

$$\begin{array}{cc} q_{\phi }\left(S_{t}|X_{1:T}\right) & =\mathrm{Cat}\left(\sigma \left(\Phi_{\phi }^{S}\left(h_{t}^{X}\right)\right)\right),\\ q_{\phi }\left(Z_{t}|X_{1:T}\right) & =\mathrm{Norm}\left(\mu_{\phi }^{Z}\left(h_{t}^{X}\right),\sigma_{\phi }^{Z}\left(h_{t}^{X}\right)\right),\\ h_{1:T}^{X} & =B-\mathrm{LSTM}(X_{1:T}), \end{array}$$

where $\Phi_{\phi }^{S}\left(\cdot \right)$, and $\mu_{\phi }^{Z}\left(\cdot \right)$ and $\sigma_{\phi }^{Z}\left(\cdot \right)$ are neural network mappings that parameterize categorical and Gaussian distributions, respectively.

#### 3.2.2. ELBO Derivation

We can derive ELBO by plugging in the generative $p_{\theta }\left(X,L,S,Z\right)$ and variational $q_{\phi }\left(S,Z|X\right)$ distributions from Equations (1) and (6) respectively into Equation (5) (subscript over *n* is dropped for brevity):

$$\begin{array}{l} |L_{n,t}(\theta ,\phi )|=\\ \;E_{q_{\phi }\left(Z_{t}|X\right)}\left[∥X_{t}-\mu_{\theta }^{X}\left(Z_{t}\right){∥}_{F}^{2}\right]+\\ \;E_{q_{\phi }\left(S|X\right)}\left[\mathrm{CELoss}\left(\sigma \left(\Phi_{\theta }^{L}\left(h_{t}^{S}\right)\right),L\right)\right]+\\ \;\sum_{s}q_{\phi }\left(S_{t-1}=s|X\right)E_{q_{\phi }\left(Z_{t-1}|X\right)}\left[\mathrm{KL}\left(q_{\phi }\left(S_{t}|X\right)||p_{\theta }\left(S_{t}|S_{t-1}=s,Z_{t-1}\right)\right)\right]+\\ \;\sum_{s}q_{\phi }\left(S_{t}=s|X\right)E_{q_{\phi }\left(Z_{t-\ell }|X\right)}[\mathrm{KL}(q_{\phi }\left(Z_{t}|X\right)∥p_{\theta }\left(Z_{t}|Z_{t-\ell },S_{t}=s\right))], \end{array}$$

where CELoss(·,·) denotes the cross-entropy loss function. The first two terms correspond to motion sequence reconstruction loss and action label prediction loss, respectively. The third and fourth terms regularize discrete and continuous latent transitions, respectively. We estimate the gradients of ELBO with respect to generative and variational parameters (θ and ϕ) using a reparameterized sample from the continuous latent $Z_{t}$ [47]. In the regularization terms of ELBO, the expectations over discrete latent $S_{t}$ are easily handled by enumerating over its *S* possible states.

### 3.3. Summary of the Proposed Method

We have visualized the framework of our model in Figure 2. As shown in this figure, our model encodes an input motion sequence $\left\{X_{1},\cdots ,X_{T}\right\}$ into a sequence of hidden features $\left\{h_{1},\cdots ,h_{T}\right\}$ using a bidirectional LSTM. The resulting hidden features are fed to two separate MLPs for estimating variational distribution parameters of discrete latents $\left\{S_{1},\cdots ,S_{T}\right\}$ and continuous latents, $\left\{Z_{1},\cdots ,Z_{T}\right\}$. These posterior distributions are then sampled to obtain their latent values. We decode to the input motion sequence $\left\{{\hat{X}}_{1},\cdots ,{\hat{X}}_{t}\right\}$ by feeding continuous latents *Z* to an MLP. We also decode to the associated action labels $\left\{{\hat{L}}_{1},\cdots ,{\hat{L}}_{T}\right\}$ by feeding probability vectors of the discrete latents S to a bidirectional LSTM. We estimate the priors for the discrete latents $p(S_{t})$ and continuous latents $p(Z_{t})$ from the values of sampled latents using two separate MLPs.

## 4. Experimental Results

We evaluated the performance of our model on two large-scale 3D skeletal motion datasets in terms of action recognition and dynamical prediction. First, we give a brief description of each skeletal dataset and the performance metrics we used throughout our experiments. Next, we introduce comparison baselines and provide implementation details of our model. Then, we describe our experimental results on each of the benchmark datasets. Finally, we conduct an ablation study to evaluate the impact of our modeling assumption. The classification and predictive performance of our model is summarized in Table 2.

### 4.1. 3D Skeletal Datasets

We evaluated our model on a large-scale action recognition dataset, NTU RGB+D 60 [9], and a benchmark dataset for 3D human sensing in natural environments, Human3.6M [48].

NTU RGB+D 60 dataset contains 56,578 3D skeletal motion sequences for 60 action classes captured from 40 subjects and recorded by three Kinect V2 cameras concurrently from different view angles. Each motion sequence contains 3D locations of D=25 skeleton joints recorded over time. Following the suggestion in [9], we split the dataset into train and test under two settings: (i) Cross-Subject (x-sub), where the subjects are split into train and test groups, yielding 40,091 and 16,487 train and test sequences, respectively, and (ii) Cross-View (x-view), where 37,646 sequences recorded from camera 2 and 3 are used for train and 18,932 sequences collected from camera 1 are used for test. We preprocessed sequences with normalization and translation following [9]. We further downsampled skeletal sequences by a factor of six (to 5 Hz) and padded all sequences to T=50 time points by repetition.

Human3.6M dataset contains 3.6 million 3D human poses from 11 professional actors in 15 different scenarios (directions, discussion, eating, sitting down, greeting, taking photo, posing, making purchases, smoking, waiting, walking, sitting, phone call, walking dog, and walking together). This dataset provides accurate 3D positions of D=17 body joints recorded with high-speed motion capture system at 50 Hz from two motion sequences per each actor and action label. However, only 3D pose data from 7 actors are provided (for training and validation) and the remaining poses for 4 actors are kept confidential for testing purposes in video-based pose estimation models. Therefore, in this paper for the purpose of action recognition we focus on the 7 actors from whom we have their 3D pose data and corresponding action labels. We select data from 5 actors (1, 5, 6, 7, and 8) for training and leave data from 2 actors (9 and 11) for test yielding 150=5×15×2 motion sequences for training and 60=2×15×2 motion sequences for test. We preprocessed sequences with normalization and translation (translating torso to origin) and further downsampled skeletal sequences by a factor of ten to 5 Hz resulting in sequences of temporal length T=100−635.

### 4.2. Performance Metric

We report test set classification accuracy for quantifying the model’s capacity for action recognition. We further report a histogram of dynamical state usage per action label and cluster actions based on their state-correlation matrix. In order to quantify the performance of our dynamical generative model, we compute its temporal predictive error on the test set. To this end, we predict the next time point on a test set using the generative model learned on our train set:

$$\begin{array}{c} {\hat{X}}_{t+1}∼p_{\theta }\left({\hat{X}}_{t+1}|{\hat{Z}}_{t+1}\right), \end{array}$$

where

$$\begin{array}{cc} {\hat{Z}}_{t+1} & ∼p_{\theta }\left({\hat{Z}}_{t+1}|Z_{t+1-\ell },{\hat{S}}_{t+1}\right),\\ {\hat{S}}_{t+1} & ∼p_{\theta }\left({\hat{S}}_{t+1}|S_{t},Z_{t}\right)\\ S_{t} & ∼q_{\phi }\left(S_{t}|X_{1:t}\right),\\ Z_{t}∼q_{\phi }\left(Z_{t}\right|X_{1:t}), & \;Z_{t+1-\ell }∼q_{\phi }\left(Z_{t+1-\ell }|X_{1:t}\right), \end{array}$$

where variables with hat denote predicted values and variables without hat are sampled from their posterior. In other words, latent values for the next time point are predicted from their historical values and then these predicted latent values are used for generating the next motion in the sequence. We then run inference on $X_{t+1}$, the actual observation at t+1, to obtain $Z_{t+1}$ and $S_{t+1}$, and add them to the historical data for prediction of the next time point ${\hat{X}}_{t+2}$ in the same way. We repeat these steps to make predictions in a rolling manner across the test set and report their normalized root-mean-square error (NRMSE%):

$$\mathrm{NRMSE}\%=\frac{∥X-\hat{X}{∥}_{F}}{{∥X∥}_{F}}\times 100,$$

where *X* and $\hat{X}$ are the ground-truth and predicted values, respectively, and ${∥\cdot ∥}_{F}$ denotes Frobenius norm. Note that this metric is related to the test-set predictive log-likelihood in our case of Gaussian distributions (with a multiplicative/additive constant).

### 4.3. Comparison Baselines

We compared our model in terms of action classification accuracy against a state-of-the-art action recognition model, Part-Aware LSTM (P-LSTM) [9], and in terms of dynamical prediction accuracy against two established Bayesian switching dynamical models, recurrent switching linear dynamical systems (rSLDS) [20] and switching linear dynamical systems (SLDS) [18], a state-of-the-art dynamical matrix factorization method, Bayesian temporal matrix factorization (BTMF) [49], which models higher-order linear dependencies, a state-of-the-art deep state-space model, recurrent Kalman networks (RKN) [32], which employs first-order nonlinear transitions, and a deep neural network forecasting method, long- and short-term time-series network (LSTNet) [35], which employs vector auto-regression.

The P-LSTM model divides the skeleton into five major groups of joints (torso, two hand, and two legs) and assigns a distinct LSTM cell for each body part. The outputs of these part-based cells are then concatenated to learn their common temporal patterns and are combined in the global level representation for final action classification. Similar to P-LSTM, we employ an LSTM structure for encoding motion dynamics, however, we additionally define a dynamical deep generative model to decode from these encoded features to the original motion sequences and their associated action labels. The use of LSTM structure in both P-LSTM and our method, makes P-LSTM a fair comparison baseline for the purpose of this work for evaluating the impact of our generative dynamical modeling assumption on action classification.

Dynamical baselines can be separated into two categories of “switching”: rSLD and SLDS, and “non-switching”: BTMF, RKN, LSTNet. rSLDS and SLDS models learn a switching dynamical model over sequential data by defining a set of discrete and continuous temporal latents similar to our method. However, they consider a first-order and linear transition model between temporal latents. On the other hand, BTMF learns dynamical transitions by defining an auto-regressive transition model on its temporal latents such that each latent is estimated from a linear combination of its preceding latents. RKN method employs a first-order transition network (parameterized by a neural network) to non-linearly map from preceding value of a temporal latent to its current value. LSTNet is a non-Bayesian deep learning-based forecasting approach which directly processes high-dimensional sequential data by employing a vector auto-regressive transition model to relate neighboring time points. Our method extends these baselines by considering a switching nonlinear auto-regressive transition model (empowered by neural networks) which is able to capture multi-modal and higher-order nonlinear dependencies in motion sequences. The aforementioned baselines share similar modeling assumptions with our method; therefore, they constitute a fair comparison.

### 4.4. Implementation Details

We implemented our model in PyTorch v1.8 [50] and run our experiments on a TITAN Xp GPU. The network architectures for all nonlinear mappings in our model are reported in Table 1. Our model has O(KD) variational and $O\left(S\left|\ell \right|K^{2}+\mathrm{KD}+\mathrm{KA}\right)$ generative parameters. We employed Adam optimizer [51] with lr = 0.01 and trained our models for 300 epochs. Our method took roughly 6.0 seconds per epoch with batch size of 3000 for NTU RGB+D dataset and 0.7 seconds per epoch with batch size of 1 for Human3.6M dataset. Each epoch took around 6 s.

### 4.5. Evaluation Results on NTU RGB+D 60 Dataset

**Figure 3:**
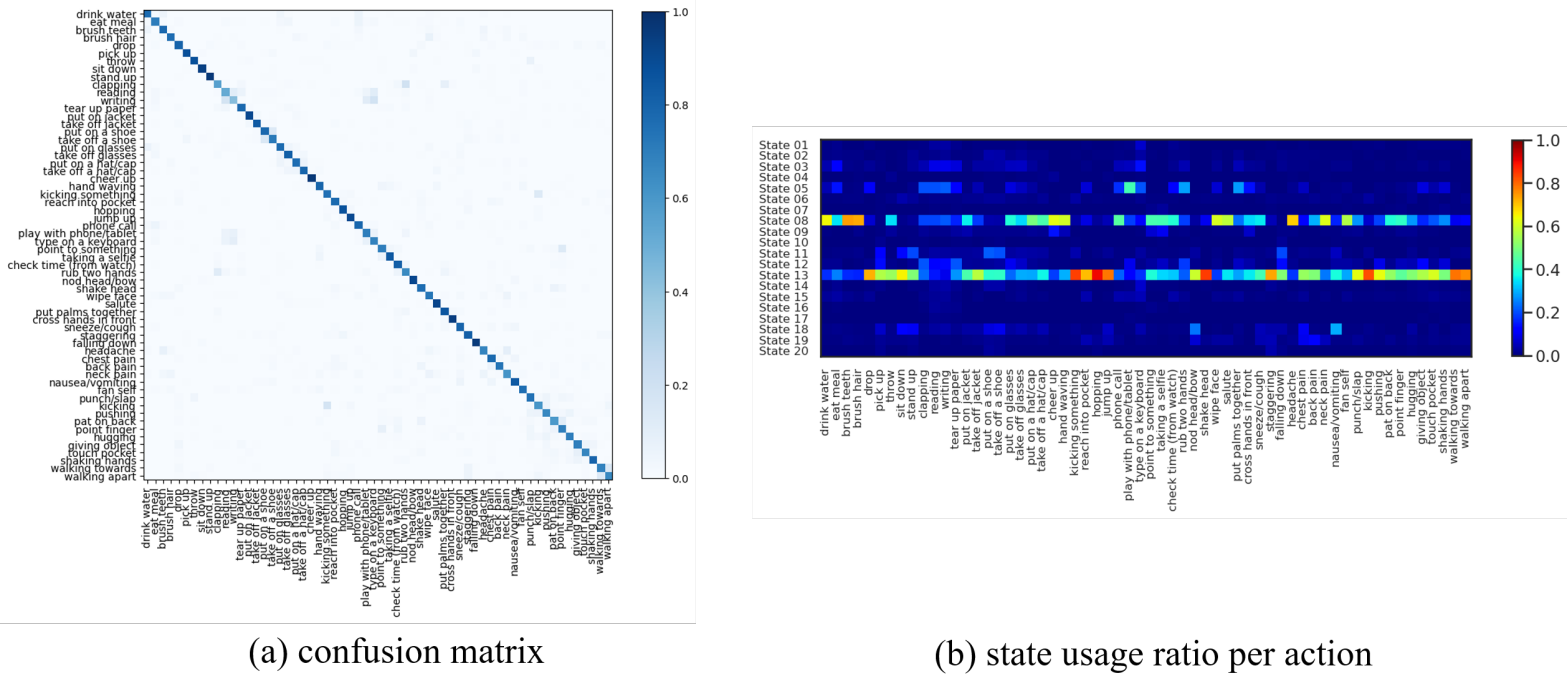
(**a**) Confusion matrix of action classification for x-view setup of NTU RGB+D 60 dataset. The model has difficulty in distinguishing {reading, writing, typing, playing with phone} or {clapping, rub two hands, put palms together} for instance. (**b**) State usage ratio per each action label shows predominant usage of state 05, state 08 and state 13 (as major motion primitives) appearing to represent hand, upper-body, and lower-body movements for most action labels, respectively.

**Figure 4:**
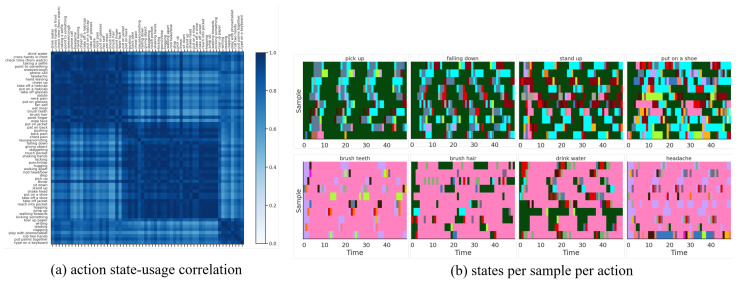
(**a**) Action correlation matrix based on state-usage for x-view setup of NTU RGB+D 60 dataset, post-processed with a spectral co-clustering algorithm, reveals three major action groups. (**b**) Inferred states over time for ten randomly-selected motion sequences from each action label of {pick up, falling down, stand up, put on a shoe} and {brush teeth, brush hair, drink water, headache} confirms similar states across similar actions.

We fit our model on this dataset with S=20 discrete states, ℓ={1,2} temporal lags (i.e., a 2nd-order Markov model), and K=15 for the continuous latent dimension. We fit P-LSTM and dynamical baselines accordingly with their default settings and match their structural hyper-parameters (if applicable) for a fair comparison. As reported in Table 2, our model outperformed P-LSTM in terms of action classification accuracy in both cross-view and cross subject setup with 76.60% and 67.52% against 70.27% and 62.93%, respectively. We have visualized the confusion matrix of this classification (for x-view setup) in Figure 3a which shows difficulty in distinguishing {reading, writing, typing, playing with phone} or {clapping, rub two hands, put palms together} for instance. We have further visualized states usage shares for each action label in Figure 3b which are mainly dominated by usage of state 05, state 08 and state 13 (as major motion primitives) appearing to represent hand, upper-body and lower-body movements, respectively, for most action labels. To further explore this, we computed action correlations in terms of their state-usage similarity, applied a spectral co-clustering algorithm [52] on the resulting correlation matrix, and visualized that in Figure 4a. This figure reveals the three major action groups based on their inferred state latents. We have visualized inferred states over time for ten randomly-selected motion sequences from each action label of {pick up, falling down, stand up, put on a shoe} and {brush teeth, brush hair, drink water, headache} in Figure 4 which confirms that similar actions share similar states as their motion primitives.

**Figure 5:**
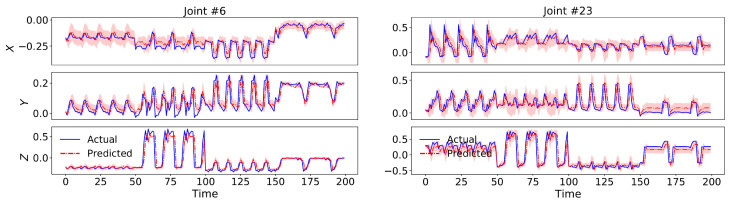
Test set predictions of four skeletal sequences from x-view setup of NTU RGB+D 60 dataset along with their uncertainty intervals for two sample body joints.

Our model also outperformed all the dynamical baselines in terms of dynamical prediction error with 17.23% and 18.34% for cross-view and cross-subject setup, respectively, by employing a 2nd-order switching nonlinear dynamical model. We have visualized test set predictions of four skeletal sequences along with their uncertainty intervals for two sample body joints in Figure 5, which indicate the capability of our model in following and predicting the actual dynamics.

### 4.6. Evaluation Results on Human3.6M Dataset

**Figure 6:**
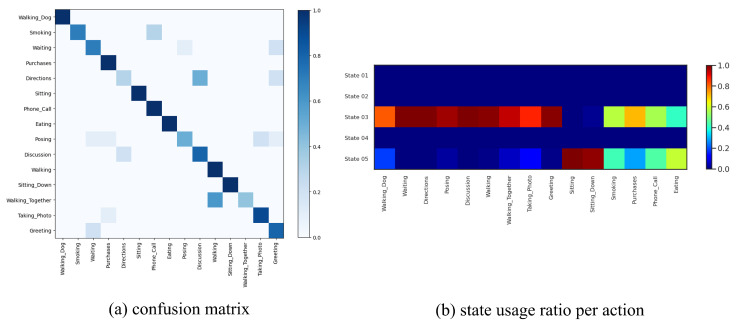
(**a**) Confusion matrix of action classification for Human3.6M dataset. The model has difficulty in distinguishing “smoking” from “phone call”, “showing directions” from “discussion”, “walking together” from “walking”, or “waiting” and “directions” from “greeting” for instance. This is expected as these actions share very similar motion patterns and are hard to determine from pose data without any visual features. (**b**) State usage histogram per each action label shows exclusive usage of state 03 and state 05 as major motion primitives which appears to represent arms and legs movements, respectively. The other three states are not utilized.

**Figure 7:**
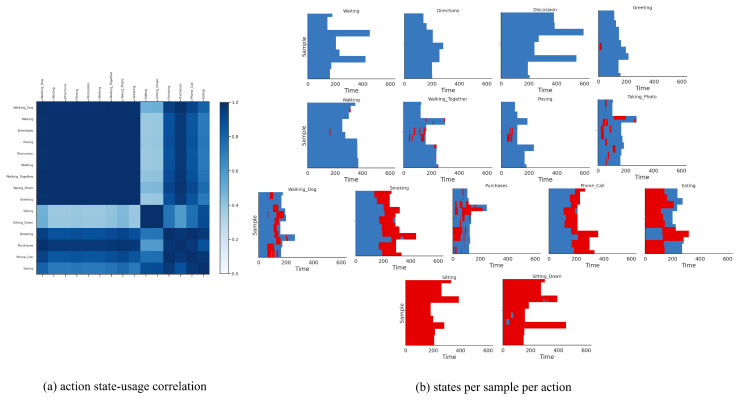
(**a**) Action correlation matrix based on state-usage for Human3.6M dataset, post-processed with a spectral co-clustering algorithm, reveals three major action groups. To be specific, actions of “waiting”, “showing directions”, “discussion”, “greeting”, “walking”, “walking together”, “posing”, “taking photos” and “walking dog” are clustered together because of their dominant usage of state 03, while actions of “sitting” and “sitting down” are clustered together because of their dominant usage of state 05. On the other hand, actions of “smoking”, “phone call”, “eating” and “making purchases” are clustered together as they use both states (03 and 05) almost equally. While these latter actions mainly involve hands, they are mostly performed in a *sitting* posture. (**b**) We have visualized inferred states over time for ten randomly-selected motion sequences from each action label which confirms similar state-usage among similar actions.

We fit our model on this dataset with S=5 discrete states, ℓ={1,2} temporal lags, and K=5 for the continuous latent dimension. We fit P-LSTM and dynamical baselines accordingly by matching their structural hyper-parameters (if applicable). As reported in Table 2, our model outperformed P-LSTM in terms of action classification accuracy with 78.33% against 71.67%, respectively. We have visualized the confusion matrix of this classification in Figure 6a which shows that the model has difficulty in distinguishing “smoking” from “phone call”, “showing directions” from “discussion”, “walking together” from “walking”, or “waiting” and “directions” from “greeting” for instance. This is expected as these actions share very similar motion patterns and are hard to determine from pose data without any visual features. We have further visualized states usage shares for each action label in Figure 6b which are exclusive to the usage of state 03 and state 05 (as major motion primitives) appearing to represent arms and legs movements, respectively (the other three states are never used). Additionally, we computed action correlations in terms of their state-usage similarity, and applied a spectral co-clustering algorithm on the resulting correlation matrix, and visualized that in Figure 7a. This figure reveals three major action groups based on their inferred state latents. To be specific, actions of “waiting”, “showing directions”, “discussion”, “greeting”, “walking”, “walking together”, “posing”, “taking photos” and “walking dog” are clustered together because of their dominant usage of state 03, while actions of “sitting” and “sitting down” are clustered together because of their dominant usage of state 05. On the other hand, actions of “smoking”, “phone call”, “eating” and “making purchases” are clustered together as they use both states (03 and 05) almost equally. While these latter actions mainly involve hands, they are mostly performed in a *sitting* posture. We have visualized inferred states over time for ten randomly-selected motion sequences from each action label in Figure 7 which confirms similar state-usage between similar actions.

**Figure 8:**
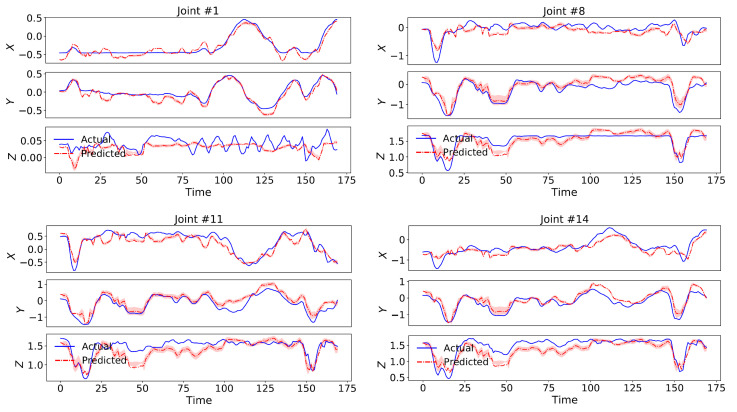
Test set predictions of a sequence in Human3.6M dataset along with its uncertainty intervals for four sample body joints, which indicate the capability of our model in following and predicting the actual dynamics.

Our model again outperformed all the dynamical baselines in terms of dynamical prediction error with 20.76% by employing a 2nd-order switching nonlinear dynamical model. We have visualized predictions of a test set sequence along with its uncertainty intervals for four sample body joints in Figure 8, which indicate the capability of our model in following and predicting the actual dynamics.

### 4.7. Ablation Study

We conducted an ablation study to evaluate the impact of motion sequence reconstruction in our model on action classification accuracy. To this end, we trained a version of our model with only discrete latents which merely regresses input sequences to their action labels. The classification accuracy of this variant are reported in Table 2 which shows a significant decrease compared to the original model. We believe that the motion reconstruction term guides the model towards learning more expressive latent features (similar to encoding in auto-encoders) which are then helpful for better decoding to the action labels.

## 5. Conclusions

We proposed a deep switching dynamical model for action recognition and dynamical prediction in 3D skeletal motion data. Our model parsed dynamical states in the low-dimensional generative process of the data. We parameterized these low-level temporal generative models with regard to a switching deep autoregressive prior to enable multimodal and higher-order dynamical estimation. Our classification and and predictive results on two large-scale 3D skeletal datasets demonstrated the superior performance of the proposed model in comparison with the state-of-the-art methods. Specifically, our method achieved higher action classification accuracies by incorporating a dynamical generative framework in comparison with a state-of-the-art model which did not model dynamics. Our model also achieved better predictive performance (in terms of next-time point prediction on the test set) when compared to the state-of-the-art dynamical prediction models by employing a nonlinear second-order dynamical transition model. Also, the sequence of discrete latents in our method provided qualitative and quantitative interpretations about motion primitives that gave rise to each action class.

For future work, we plan to replace the LSTM structure by a graph convolutional neural network to better capture spatial correlations that are present in skeletal data by explicitly incorporating joint-hierarchy information. There are still several limitations with our method and previous works which need to be addressed in future. A major limitation with our method and previous works in human action recognition is that (I) they are not directly generalizable to new action classes and need re-training. (II) As with other sequence modeling methods, our model can be sensitive to temporal sampling frequency. (III) Since our method processes joint locations, it is not invariant to the rotations of skeletal data and may not generalize well on globally rotated data. This is also the case with a vast body of previous works. (IV) Although our method demonstrates an improved prediction error over state-of-the-art dynamical models, its predictions may still diverge over longer horizons due to accumulation error.

## Figures and Tables

**Table 1:** Network architectures for the nonlinear mappings in our generative model (gModel) and inference model (iModel).

**gModel**	$\Phi_{\theta }^{s}:R^{K}\to R^{S}$	${\left(\mu ,\sigma \right)}_{\theta }^{Z,s}:R^{|\ell |\times K}\to R^{K,K}$	$\Phi_{\theta }^{L}:R^{2K}\to R^{A}$	$\mu_{\theta }^{X}:R^{K}\to R^{3D}$
Input	$Z_{t-1}\in R^{K}$	$Z_{t-\ell }\in R^{|\ell |\times K}$	$h_{t}^{S}\in R^{2K}$	$Z_{t}\in R^{K}$
1	FC K×K ReLU	FC |ℓ|×K×K ReLU	FC 2K×K ReLU	FC K×2K ReLU
2	FC K×K ReLU	AvgPool(|ℓ|)	FC K×A	FC 2K×2K ReLU
3	FC K×S	FC K×K ReLU		FC 2K×2K ReLU
4		FC K×(K+K)		FC 2K×3D
**iModel**	$\Phi_{\phi }^{S}:R^{2K}\to R^{S}$		${\left(\mu ,\sigma \right)}_{\phi }^{Z}:R^{2K}\to R^{K,K}$	
Input	$h_{t}^{X}\in R^{2K}$		$h_{t}^{X}\in R^{2K}$	
1	FC 2K×2K ReLU		FC 2K×2K ReLU	
2	FC 2K×2K ReLU		FC 2K×2K ReLU	
3	FC 2K×S		FC 2K×(K+K)	

**Table 2:** Comparison of action classification accuracy and dynamical prediction error. Our model outperformed all the baselines.

		Classification Accuracy (%)			Dynamical Prediction (NRMSE%)					
	**Model**	**Ours**	**P-LSTM**	**Ablation**	**Ours**	**rSLDS**	**SLDS**	**BTMF**	**RKN**	**LSTNet**
**Dataset**				**Ours**						
NTU (x-view)		**76.60**	70.27	69.81	**17.23**	22.45	22.19	18.81	22.64	20.22
NTU (x-sub)		**67.52**	62.93	60.74	**18.34**	23.68	25.49	21.86	25.63	24.15
Human3.6M		**78.33**	71.67	73.33	**20.76**	27.99	28.57	23.83	24.04	23.12

The best results are highlighted in bold fonts.

## Data Availability

Restrictions apply to the availability of these data. Data were obtained from ROSE Lab datasets and Human3.6M dataset and are available at rose1.ntu.edu.sg/dataset/ and vision.imar.ro/human3.6m/, respectively, with their permissions.
